# Identification of two flavonoids antiviral inhibitors targeting 3C-like protease of porcine epidemic diarrhea virus

**DOI:** 10.3389/fmicb.2024.1357470

**Published:** 2024-03-20

**Authors:** Zhonghua Li, Liangyun Zhu, Lu Wang, Yizhi Huang, Yi Zhang, Di Zhao, Lei Wang, Dan Yi, Yongqing Hou, Tao Wu

**Affiliations:** Hubei Key Laboratory of Animal Nutrition and Feed Science, Wuhan Polytechnic University, Wuhan, China

**Keywords:** porcine epidemic diarrhea virus, 3C-like protease inhibitor, flavonoid, antiviral, baicalein, baicalin

## Abstract

Porcine epidemic diarrhea virus (PEDV) has caused severe damage to the global pig industry in the past 20 years, creating an urgent demand for the development of associated medications. Flavonoids have emerged as promising candidates for combating coronaviruses. It is believed that certain flavonoids can directly inhibit the 3C-like protease (3CL^pro^), thus displaying antiviral activity against coronaviruses. In this investigation, we applied a flavonoid library to screen for natural compounds against PEDV 3CL^pro^. Baicalein and baicalin were found to efficiently inhibit PEDV 3CL^pro^*in vitro*, with the IC_50_ value of 9.50 ± 1.02 μM and 65.80 ± 6.57 μM, respectively. A docking analysis supported that baicalein and baicalin might bind to the active site and binding pocket of PEDV 3CL^pro^. Moreover, both baicalein and baicalin successfully suppressed PEDV replication in Vero and LLC-PK1 cells, as indicated by reductions in viral RNA, protein, and titer. Further investigation revealed that baicalein and baicalin mainly inhibited the early viral replication of the post-entry stage. Furthermore, baicalein showed potential effects on the attachment or invasion step of PEDV. Collectively, our findings provide experimental proof for the inhibitory effects of baicalein and baicalin on PEDV 3CL^pro^ activity and PEDV infection. These discoveries may introduce novel therapeutic strategies for controlling porcine epidemic diarrhea (PED).

## 1 Introduction

Porcine epidemic diarrhea (PED) caused by porcine epidemic diarrhea virus (PEDV) is a highly contagious intestinal infectious disease of pigs, and its characteristic clinical symptoms are vomiting, diarrhea and dehydration of the infected piglets (Jung et al., 2020). The primary consequence of this disease is the extremely high mortality rate, which can reach 100% for piglets under the age of 7 days. The disease was first reported in the United Kingdom in 1971 (Wood, 1977) and has since become widespread in Europe (Debouck and Pensaert, 1980), as well as in some Asian countries such as Japan (Takahashi et al., 1983), South Korea (Chae et al., 2000), China (Chen et al., 2008) and Thailand (Puranaveja et al., 2009). Over the past two decades, worldwide outbreaks of PED caused by PEDV variant strains have resulted in significant losses for the global pig industry (Zhang et al., 2023). Vaccination is regarded as an efficient measure to control PED. However, vaccine immunization often fails because of the high mutation rate of PEDV, and is ineffective in piglets that have already been infected. Consequently, there is an urgent need to develop effective therapies to combat PEDV infection.

PEDV is classified as an Alphacoronavirus genus within the Coronaviridae family (Pensaert and de Bouck, 1978; Cavanagh, 1997). Similar to other members of the coronavirus family, PEDV possesses an RNA genome that encodes two polyproteins: pp1a and pp1ab. These polyproteins can be cleaved into 16 mature non-structural proteins (NSPs) through the action of two viral proteases (Ziebuhr et al., 2000; Thiel et al., 2001; Lee, 2016; Lin and Zhang, 2022). One of these viral proteases is known as the 3C-like protease (3CL^pro^), which is encoded by the non-structural gene 5. The primary function of 3CL^pro^ is to cleave the 11 conserved sites within the polyproteins (Ye et al., 2016). 3CL^pro^ plays significant roles in coronavirus infection, as the mature NSPs directly participate in the replication of the PEDV genome and the immune escape process (Wang et al., 2015). Moreover, the high sequence similarity of 3CL^pro^ among different coronaviruses makes it an ideal target for broad-spectrum anti-coronavirus treatments.

Flavonoids, a class of polyphenolic-plant secondary metabolites, are naturally occurring compounds found in fruits, vegetables, and certain beverages (Cushnie and Lamb, 2005; Badshah et al., 2021). Flavonoids have been reported to possess various beneficial properties such as antioxidant activity (Pietta, 2000), anti-inflammatory effects (Maleki et al., 2019), antimutagenic properties (Okuno et al., 2019), anticancer potential (Kopustinskiene et al., 2020), and antiviral activity (Ninfali et al., 2020). Previous studies have demonstrated that flavonoids can exert their effects at different stages of viral infection, including viral entry, genome replication, and protein translation. In the case of coronaviruses, numerous studies have shown that certain flavonoids can inhibit viral replication by targeting the coronavirus 3CL^pro^ (Jo et al., 2020).

In this study, a proteolytic method was employed to screen PEDV 3CL^pro^ inhibitors from a flavonoid library. Baicalein and baicalin were found to significantly inhibit PEDV 3CL^pro^ activity and PEDV replication *in vitro*. The results provide novel insights into the potential use of baicalein and baicalin as anti-PEDV drugs, which might be beneficial for addressing the current problem caused by PED.

## 2 Materials and methods

### 2.1 Viruses, cells and flavonoid library

The PEDV YN13 (GenBank accession No. KT021228), a PEDV variant strain, was isolated from the intestine of a piglet with severe diarrhea and was passaged for 13 generations. The DR13-GFP strain was a recombinant strain which was constructed by replacing the ORF3 gene of the DR13 strain (GenBank accession no. JQ023161) with the GFP gene using the reverse genetic technique. Vero and LLC-PK1 cells were cultured in Dulbecco’s modified Eagle’s medium (DMEM) containing 8% fetal bovine serum (FBS) and were incubated at 37°C with 5% CO_2_. The flavonoid library containing 270 compounds (MedChemExpress, Shanghai, China) was used for screening for the inhibitors of PEDV 3CL^pro^. Detailed information on the flavonoid library is shown in the Supplementary File S1.

### 2.2 Protein expression and purification

The PEDV 3CL^pro^ expression vector was obtained by cloning the PEDV nsp5 gene into the Pet-28a (+) vector. This vector was then transformed into the *E.coli BL21* (DE3) strain to create recombinant bacteria. The bacteria were cultured at 37°C in Luria Bertani medium until the optical density value at a wavelength of 600 nm (OD_600_) reaching 0.6–0.8. After that, 0.8 mM isopropyl β-D-thiogalactoside (IPTG) was added to induce protein expression. The bacteria were harvested after incubation at 37°C for 24 h, resuspended in PBS and disrupted. The supernatant was filtered and loaded onto a nickel iminodiacetic acid NUPharose fast flow (Nuptec, Hangzhou, China) to purify the protein. Subsequently, the N and C terminal His-tagged protein was eluted using elution buffer. The protein eluent was desalted by dialysis in PBS buffer and was concentrated by ultrafiltration.

### 2.3 Fluorescence resonance energy transfer (FRET) assays

In order to evaluate the enzymatic activity of the purified protease, a peptide substrate whose sequence is Dabcyl-YNSTLQ↓AGLRKM-E-Edans was synthesised based on the cleavage site of PEDV 3CL^pro^. The two fluorophores, namely Dabcyl and Edans, formed a quenching pair and exhibited FRET within the peptide. The enzyme reaction system (100 μL) used to measure PEDV 3CL^pro^ activity consisted of 10 μM FRET substrate, 20 mM Tris/HCl buffer (pH 7.5), and various concentrations of the purified 3CL^pro^. Fluorescence emitted by the reactions was continuously monitored for a duration of 90 min, with excitation at 340 nm and emission at 485 nm, using fluorescence plate reader reactions with a multimode reader platform (Molecular Devices, San Jose, CA, United States). For the purpose of screening PEDV 3CL^pro^ inhibitors, reaction systems (100 μL) were prepared, utilizing 10 μM FRET substrate, 20 μg/mL 3CL^pro^, 20 μM natural molecule, and 20 mM Tris/ HCl buffer (pH 7.5). Each natural molecule presented in the flavonoid library was initially incubated with 3CL^pro^ for a period of 30 min at a temperature of 37°C. Subsequently, the substrate was added to the mixture and a reaction system without any natural molecule was set up as a control group. The fluorescence of reactions was monitored according to the method mentioned above. The inhibition ratio was determined by employing the following equation: Percentage of inhibition (%) = 100× [1 − fluorescence of the experimental group (90–0 min)/fluorescence of the control group (90–0 min)].

### 2.4 Docking of PEDV 3CL^pro^ and the screened inhibitors

The crystal structures of the chosen compounds (baicalein and baicalin) as well as PEDV 3CL^pro^ (PDB: 4XFQ) were downloaded from the website https://www.rcsb.org/. Autodock 4 software was used to generate the homology models of PEDV 3CL^pro^ with the selected molecules. Grid maps of 19 × 19 × 19 grid points (center: x = 20.254, y = 11.946, z = 5.239; spacing = 1.000) were produced, covering the active site and the binding pocket of PEDV 3CL^pro^. To ensure accurate docking, the following parameters were set: a population size of 150, a maximum of energy evaluations of 5,000,000, a maximum of generations of 27,000, a mutation rate of 0.02, and a crossover rate of 0.8. Ultimately, a total of 10 docked conformations were produced and the conformation displaying the lowest binding energy was selected for the analysis of the interaction between the chosen compounds and PEDV 3CL^pro^. PyMOL software was used to analyze the docking results to obtain the most likely binding pattern.

### 2.5 Molecular dynamics (MD) simulations

The MD simulations of PEDV 3CL^pro^-baicalein and PEDV 3CL^pro^-baicalin complexes were carried out at 100 ns using GROMACS version 2018.1 (Páll et al., 2020). PRODRG was utilized to generate the ligand topology, while the protein topology was created using pdb2gmx. Solvation was carried out utilizing the simple point charge water model. In order to produce the topologies for complexes formed by proteins and ligands, the topologies of the proteins and ligands were combined. Boxes with a cube shape were generated and the complexes were positioned inside them. The electroneutrality of the system was maintained by incorporating Na^+^ and Cl^−^ ions. Additionally, the steepest descent minimization algorithm was employed to accomplish energy minimization of both the protein and protein-ligand complexes. Equilibration of the systems was also achieved through NPT and NVT simulations. Finally, all systems underwent a simulation time of 100 ns, with coordinates saved at 2 fs intervals. The trajectories of the protein and protein-ligand complexes were assessed for structural stability analysis using root-mean-square deviation (RMSD).

### 2.6 Cytotoxicity assay

To measure the cytotoxicity of baicalein and baicalin on Vero and LLC-PK1 cells, a Cell Counting Kit-8 (Beyotime Biotechnol, Shanghai, China) was utilized. To initiate the experiment, cells were seeded in a 96-well plate and were allowed to incubate at a temperature of 37°C with 5% CO_2_ until a complete monolayer was formed. Subsequently, the cells were treated with baicalein or baicalin at different concentrations at 37°C with 5% CO_2_ for 48 h. One hundred microliter of 10% CCK-8 solution (10% CCK-8 reagent in DMEM) was added to each well after being washed three times with PBS. The blank wells with 10% CCK-8 solution were set as negative control group. After 2 h in the incubator, the optical density values in each well were measured using a microplate reader at a wavelength of 450 nm (OD_450_). Cell viability was determined using the formula: cell viability = [As-Ab]/[Ac-Ab]. The OD_450_ value of the wells in the experimental groups (containing baicalein or baicalin treated cells and CCK8 solution) was denoted as As. The OD_450_ value of the mock wells (containing only CCK8 solution) was represented as Ab. Finally, the OD_450_ value of the wells in control group (containing cells and CCK8 solution) was denoted as Ac.

### 2.7 Real-time quantitative reverse transcription PCR (RT-PCR)

For YN13 strain, the cells (Vero and LLC-PK1) were grown to approximately 90% confluence in 12-well culture plates. Then the cells were infected with PEDV YN13 strain at a multiplicity of infection (MOI) of 0.001 with 8 μg/mL trypsin in the presence of baicalein or baicalin of different concentrations (0 μM, 2.5 μM, 5 μM, 10 μM, 20 μM and 40 μM for the Vero cells; 0 μM, 5 μM, 10 μM and 20 μM for the LLC-PK1 cells) for 24 h; For the DR13-GFP strain, Vero cells were grown to approximately 90% confluence in 12-well culture plates. Then the cells were infected with the DR13-GFP strain at a MOI of 0.001 in the presence of baicalein or baicalin of different concentrations (0 μM, 1.25 μM, 2.5 μM, 5 μM, 10 μM, 20 μM and 40 μM) for 36 h.

Then, the cells were collected and subjected to extract RNA using RNAiso Plus Reagent (TakaRa, Tokyo, Japan) following the manufacturer’s instructions. The cDNA was obtained by RT-PCR using the Primescript™ RT reagent Kit With gDNA Eraser (TakaRa, Tokyo, Japan) according to the manufacturer’s instructions. The Real-Time RT-PCR was performed using the SYBR Premix ExTaq (TakaRa, Tokyo, Japan) on an Applied Biosystems 7,500 Fast Real-Time PCR System (Life technologies, Marsiling, Singapore). Porcine ribosomal protein L4 (*RPL4*) and *homo sapiens* hypoxanthine phosphoribosyltransferase 1 (*HPRT1*) were used as the housekeeping gene for LLC-PK1 and Vero cells, respectively. The change of PEDV M gene expression was calculated using the 2^−ΔΔCT^ method. Primers used in the present study were listed in Table 1.

**Table 1:** Primers used in this study.

Gene		Sequence
*HPRT1* (Vero)	Forword	5’-AACCTTGCTTTCCTTGGTCA-3’
	Reverse	5’-TCAAGGGCATAGCCTACCAC-3’
*RPL19* (LLC-PK1)	Forward	5’-AACTCCCGTCAGCAGATCC-3’
	Reverse	5’-AGTACCCTTCCGCTTACCG-3’
*PEDV M*	Forward	5’-TCCCGTTGATGAGGTGAT-3’
	Reverse	5’-AGGATGCTGAAAGCGAAAA-3’

### 2.8 Tissue culture infectious dose 50 (TCID_50_) assay

For YN13 strain, the cells (Vero and LLC-PK1) were grown to approximately 90% confluence in 12-well culture plates. Then the cells were infected with PEDV YN13 strain at a MOI of 0.001 with 8 μg/mL trypsin in the presence of baicalein or baicalin of different concentrations (0 μM, 2.5 μM, 5 μM and 10 μM for the Vero cells; 0 μM, 5 μM, 10 μM and 20 μM for the LLC-PK1 cells) for 30 h; For DR13-GFP strain, Vero cells were grown to approximately 90% confluence in 12-well culture plates. Then the cells were infected with the DR13-GFP strain at a MOI of 0.001 in the presence of baicalein or baicalin of different concentrations (0 μM, 2.5 μM, 5 μM and 10 μM) for 36 h. Then the cells and the supernatant were collected and placed in −80°C. After repeated freezing and thawing three times, the supernatant was harvested by centrifuge at 12,000 rpm for 10 min at 4°C and then used for TCID_50_ assays according to the following steps.

Vero cells seeded in 96-well plates were cultured incubated at 37°C with 5% CO_2_ until the monolayer was covered. Virus samples were 10-times serially diluted before they were inoculated on the confluent cell monolayers. DR13-GFP strain samples were diluted with DMEM, while YN13 strain samples were diluted with DMEM containing 8 μg/mL of trypsin. Eight wells were inoculated with 100 μL of each dilution, and plates were incubated at 37°C with 5% CO_2_ for 2 days. For the YN13 strain, wells with syncytium formation, the specific cytopathic effect (CPE) of YN13 infection, were identified as PEDV-positive. For the DR13-GFP strain, wells with green signal under a fluorescence microscope were considered as PEDV-positive. The titration was calculated in the form of TCID_50_ using the Reed–Muench method established by Reed and Muench (1938).

### 2.9 Indirect immunofluorescence assay (IFA)

Vero cells were grown to approximately 90% confluence in 48-well culture plates. Then the cells were infected with PEDV YN13 strain at a MOI of 0.001 with 8 μg/mL trypsin in the presence of baicalein or baicalin of different concentrations (0 μM, 1.25 μM, 2.5 μM and 5 μM) for 24 h. After that, cells were gently rinsed with PBS and then fixed with 4% paraformaldehyde at room temperature for a duration of 10 min. The fixed cells underwent three subsequent washes with PBS, followed by treatment with pre-chilled methanol at a temperature of −20°C for a period of 15 min. Blocking was carried out by treating the cells with a PBS buffer that contained 5% bovine serum albumin (BSA) for 30 min at room temperature. The cells were then subjected to an incubation period of 1 h at 37°C with the PEDV S protein monoclonal antibody (diluted 1:500 in PBS), followed by three PBS washes. Afterwards, the cells were incubated at 37°C for 45 min with the Alexa 488-labeled anti-mouse antibody (Antgene, Wuhan, China). DAPI dihydrochloride (Beyotime Biotechnol, Shanghai, China) solutions were added to each well to stain the cell nuclei at room temperature for 5 min. After being washed three times with PBS, the results of IFA were observed and acquired using a fluorescence microscope.

### 2.10 Fluorescence observation of DR13-GFP infected cells

Vero cells were grown to approximately 90% confluence in 48-well culture plates. Then the cells were infected with PEDV DR13-GFP strain at a MOI of 0.001 in the presence of baicalein or baicalin of different concentrations (0 μM, 2.5 μM, 5 μM and 10 μM) for 36 h. After that, cells were gently rinsed with PBS and then incubated with hoechst 33258 (Beyotime Biotechnol, Shanghai, China) solution at 37°C for 5 min to stain the cell nucleus. After being washed three times with PBS, the GFP-expression were observed and acquired using a fluorescence microscope.

### 2.11 Time-of-addition assays

Vero cells were infected with the PEDV YN13 strain at a MOI of 0.001. Then, 20 μM of baicalein and baicalin were added into the culture medium at different time periods of PEDV infection: −2–24 h, 0–24 h, 2–24 h, 4–24 h and 6–24 h. At 24 h post infection, RNA was extracted from the cells and PEDV M gene was detected using real-time quantitative RT-PCR.

### 2.12 Statistical analysis

The half maximal inhibitory concentration (IC_50_) was determined using SPSS 17.0 software. The experiments were conducted in triplicate, and the results were presented as means ± standard deviation (SD). To assess the statistical significance, student’s t-test was performed using SPSS 17.0 software. *, *p* < 0.05 (considered significant compared with control group); **, *p* < 0.01 (considered highly significant); ***, *p* < 0.001 (considered extremely significant).

## 3 Results

### 3.1 Screening of PEDV 3CL^pro^ inhibitors from a flavonoids library

**Figure 1:**
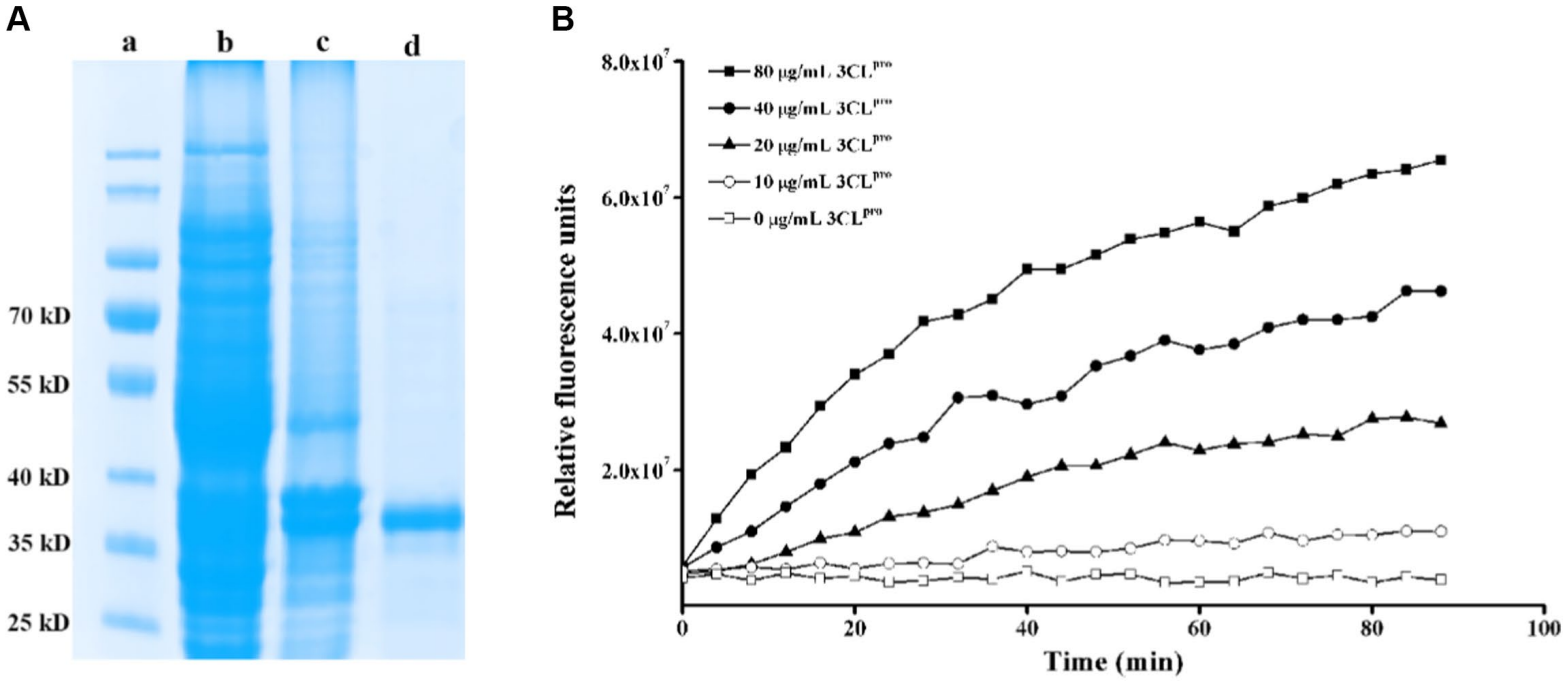
Construction of a FRET assay for detecting PEDV 3CL^pro^ cleavage activity. **(A)** Expression and purification of PEDV 3CL^pro^. Lane a, protein marker; lane b, inclusion body proteins; lane c, supernatant proteins; lane d, purified HIS-tag PEDV 3CL^pro^; **(B)** Analysis of the activity of the purified PEDV 3CL^pro^ by the FRET assay.

PEDV 3CL^pro^ was expressed using a prokaryotic expression system and was purified through affinity chromatography using a nickel column purification column. As shown in Figure 1A, PEDV 3CL^pro^ was expressed in both the supernatant and the inclusion bodies. The supernatant was then subjected to affinity chromatography, resulting in the production of a relatively pure protein after purification. To assess the cleavage activity of the purified protein, a fluorogenic peptide substrate was designed based on the conserved cleavage site recognized by PEDV 3CL^pro^ and was introduced in the FRET assays. The fluorescence increased in a time and dose-dependent manner, indicating that the purified protein effectively cleaved the fluorogenic peptide substrate (Figure 1B). These findings suggest that the constructed FRET assay can be utilized to test the enzymatic activity and to screen for the inhibitors of PEDV 3CL^pro^. A flavonoid library was employed for screening PEDV 3CL^pro^ inhibitors, and the molecules with inhibitory rates above 30% are presented in Table 2.

**Table 2:** The screened molecules and their inhibitory effect on PEDV 3CL^pro^.

Number	Name	Inhibitory rate
1	(−)-Gallocatechin gallate	97.65 ± 0.35
2	Fisetin	96.80 ± 2.64
3	Quercetagetin	91.83 ± 2.74
4	Daidzein	91.26 ± 0.85
5	Baicalein	85.62 ± 4.43
6	Myricetin	75.26 ± 2.33
7	Isorhoifolin	74.69 ± 3.49
8	4,4’-Dimethoxychalcone	68.85 ± 22.44
9	Okanin	67.96 ± 7.86
10	Schaftoside	67.55 ± 2.52
11	Nevadensin	66.93 ± 2.52
12	Dihydromyricetin	63.97 ± 4.37
13	7-Hydroxy-4H-chromen-4-one	57.58 ± 5.80
14	Neodiosmin	53.04 ± 13.13
15	4′,5-Dihydroxyflavone	52.89 ± 8.10
16	(E)-Flavokawain A	52.63 ± 9.18
17	5,7-Dihydroxychromone	51.27 ± 6.38
18	Isosinensetin	49.73 ± 6.40
19	Calycosin	49.47 ± 8.69
20	Flavone	49.39 ± 10.29
21	Mulberrin	49.34 ± 10.97
22	4-Hydroxylonchocarpin	47.25 ± 10.36
23	Narcissin	46.02 ± 15.15
24	Loureirin B	45.98 ± 3.63
25	Scutellarein	45.85 ± 7.49
26	Diosmin	44.15 ± 6.89
27	Liquiritigenin	42.66 ± 10.22
28	Genistein	42.12 ± 4.23
29	Baicalin	41.77 ± 1.50
30	Pinostrobin	41.32 ± 13.16
31	Isoliquiritin apioside	38.48 ± 11.34
32	Panasenoside	38.02 ± 14.54
33	Luteolinidin	37.68 ± 9.14
34	Neoliquiritin	37.52 ± 12.64
35	Rhamnetin	37.13 ± 11.82
36	Isosilybin	36.12 ± 10.48
37	Genistin	35.29 ± 9.37
38	Procyanidin A1	34.36 ± 11.19
39	Amentoflavone	33.68 ± 3.76

### 3.2 Inhibition of PEDV 3CL^pro^ enzymatic activity by baicalein and baicalin

**Figure 2:**
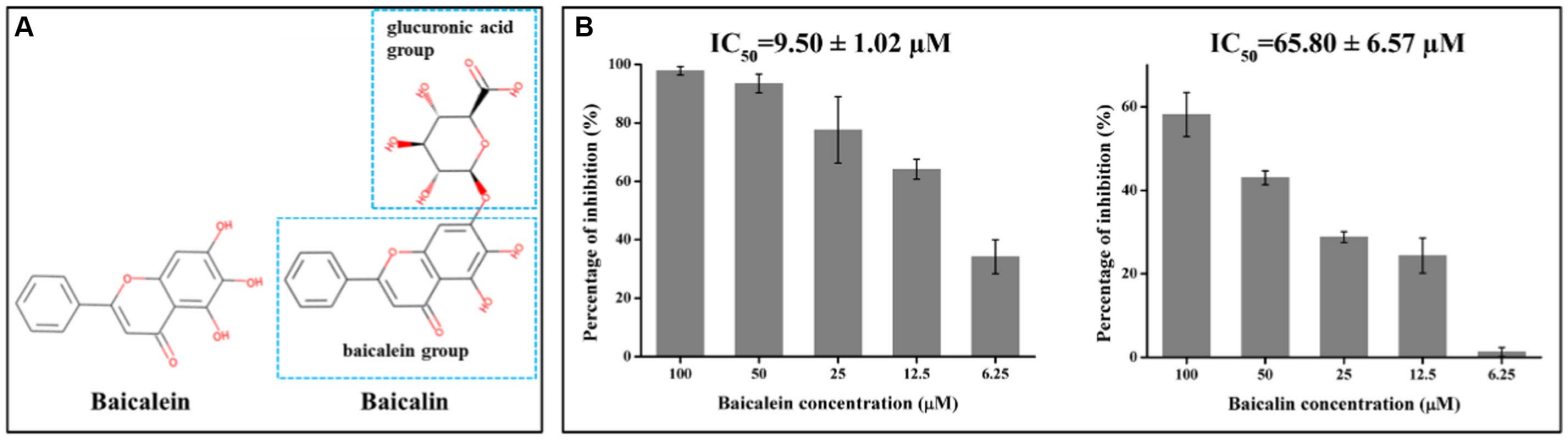
Baicalein and baicalin efficiently inhibit PEDV 3CL^pro^ activity in a dose dependent manner. **(A)** Chemical structures of baicalein and baicalin; **(B)** The inhibitory effects of baicalein and baicalin on PEDV 3CL^pro^. The IC_50_ was calculated according to the inhibition ratio for 3CL^pro^ activity of baicalein and baicalin at different concentrations using the SPSS 17.0 software. Data are shown as the mean ± SD of three independent experiments, with the error bars representing the standard deviation.

Since our aim was to search for natural molecules to control PED in the pig industry, the relatively low-cost molecules were selected to be used in the following studies. As a result, baicalein and baicalin were chosen. These two flavonoids have structural similarities, with baicalin consisting of a baicalein group and a glucuronic acid group (Figure 2A). To confirm their inhibitory effects on PEDV 3CL^pro^, different concentrations of the molecules were added to the FRET systems for detecting PEDV 3CL^pro^ enzymatic activity. The results indicated that baicalein and baicalin could inhibit PEDV 3CL^pro^ enzymatic activity in a dose-dependent manner (Figure 2B). The IC_50_ for baicalein and baicalin were found to be 9.50 ± 1.02 μM and 65.80 ± 6.57 μM, respectively.

### 3.3 Potential sites of PEDV 3CL^pro^ binding baicalein and baicalin

**Figure 3:**
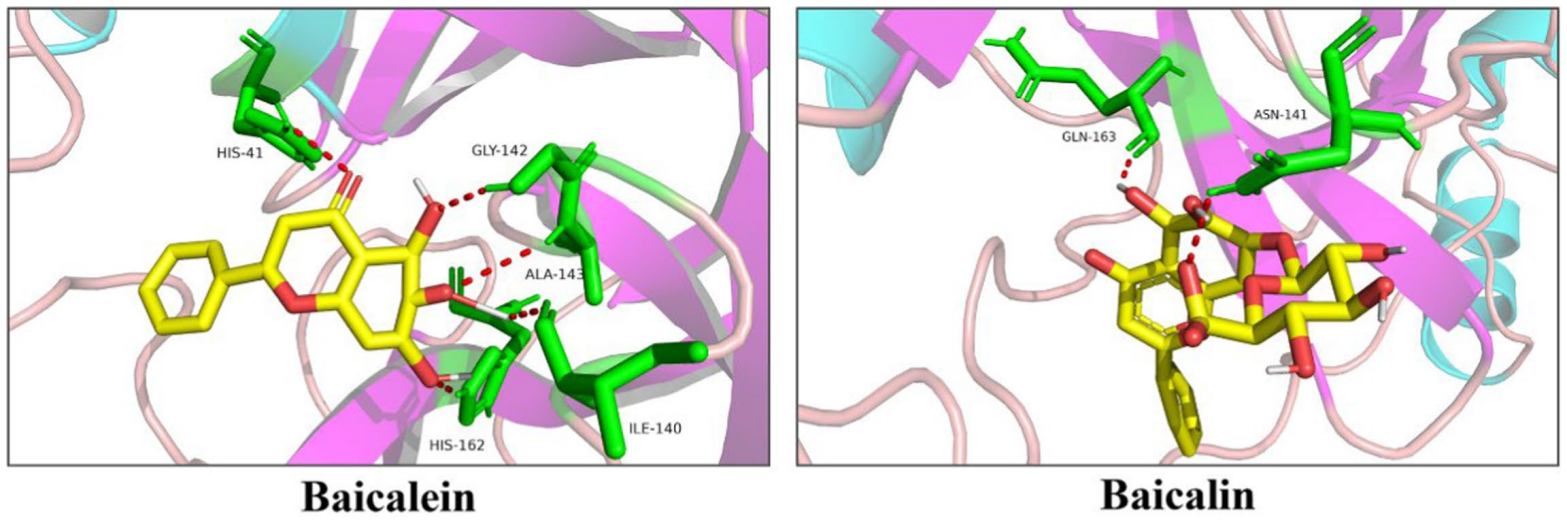
Docking of baicalein or baicalin with PEDV 3CL^pro^. Baicalein and baicalin are represented as sticks with the yellow color representing the C atom and the red color representing the O atom. The amino acid residues contacting baicalein or baicalin are represented as green sticks. Moreover, along with the bond distance, hydrogen bond interactions are shown as red dashed lines between the respective donor and acceptor atoms.

The interaction between each selected molecule and PEDV 3CL^pro^ was analyzed using molecular simulation techniques. As shown in Figure 3, baicalein could interact with His162, Ile140, Gly142, Ala143, and His41 of PEDV 3CL^pro^, while baicalin could only interact with Asn141 and Glu165.

### 3.4 MD simulations of PEDV 3CL^pro^ with baicalein and baicalin

**Figure 4:**
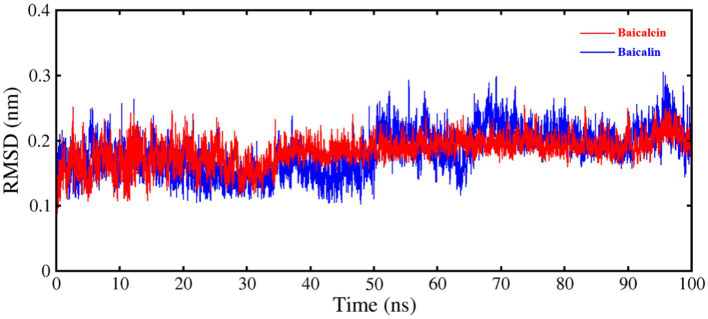
RMSD of C–Cα–N backbone for PEDV 3CL^pro^ in complex with baicalein and baicalin.

To verify the findings of the docking results and gain a deeper understanding of the stability of the ligand-protein complex, MD simulations were conducted for each complex formed by PEDV 3CL^pro^ and its inhibitors (baicalein and baicalin). The results of the MD simulations were analyzed based on the RMSD. Figure 4 clearly demonstrated that both two systems maintained equilibrium throughout the entire simulation duration, with particular stability observed in the PEDV 3CL^pro^-baicalein complex. The average RMSD values for the PEDV 3CL^pro^-baicalein and PEDV 3CL^pro^-baicalin complexes were calculated as 0.1829 nm and 0.1824 nm, respectively. Since the fluctuation amplitude of the RMSD curve was inversely related to the stability of the protein, the above results indicated that the complexes formed by PEDV 3CL^pro^ with baicalein and baicalin had high stability.

### 3.5 Toxicity of baicalein and baicalin on cells

**Figure 5:**
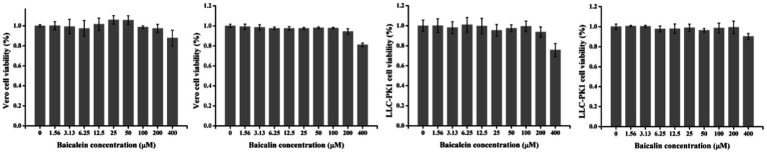
The cytotoxicity of baicalein and baicalin on Vero or LLC-PK1 cells. All the values were normalized to the cell control, which represents 100% cell viability. Data are shown as the means ± standard deviation (SD), with the error bars representing the standard deviations.

In order to investigate the effects of baicalein and baicalin on PEDV infection, it was necessary to determine the safe concentrations of them for the host cells. The viability of Vero or LLC-PK1 cells was assessed after treatment with different concentrations of baicalein or baicalin. As shown in Figure 5, the cell viability was above 95% when treated Vero and LLC-PK1 cells with baicalein and baicalin at a concentration of no more than 200 μM.

### 3.6 The anti-PEDV effects of baicalein and baicalin in Vero cells

**Figure 6:**
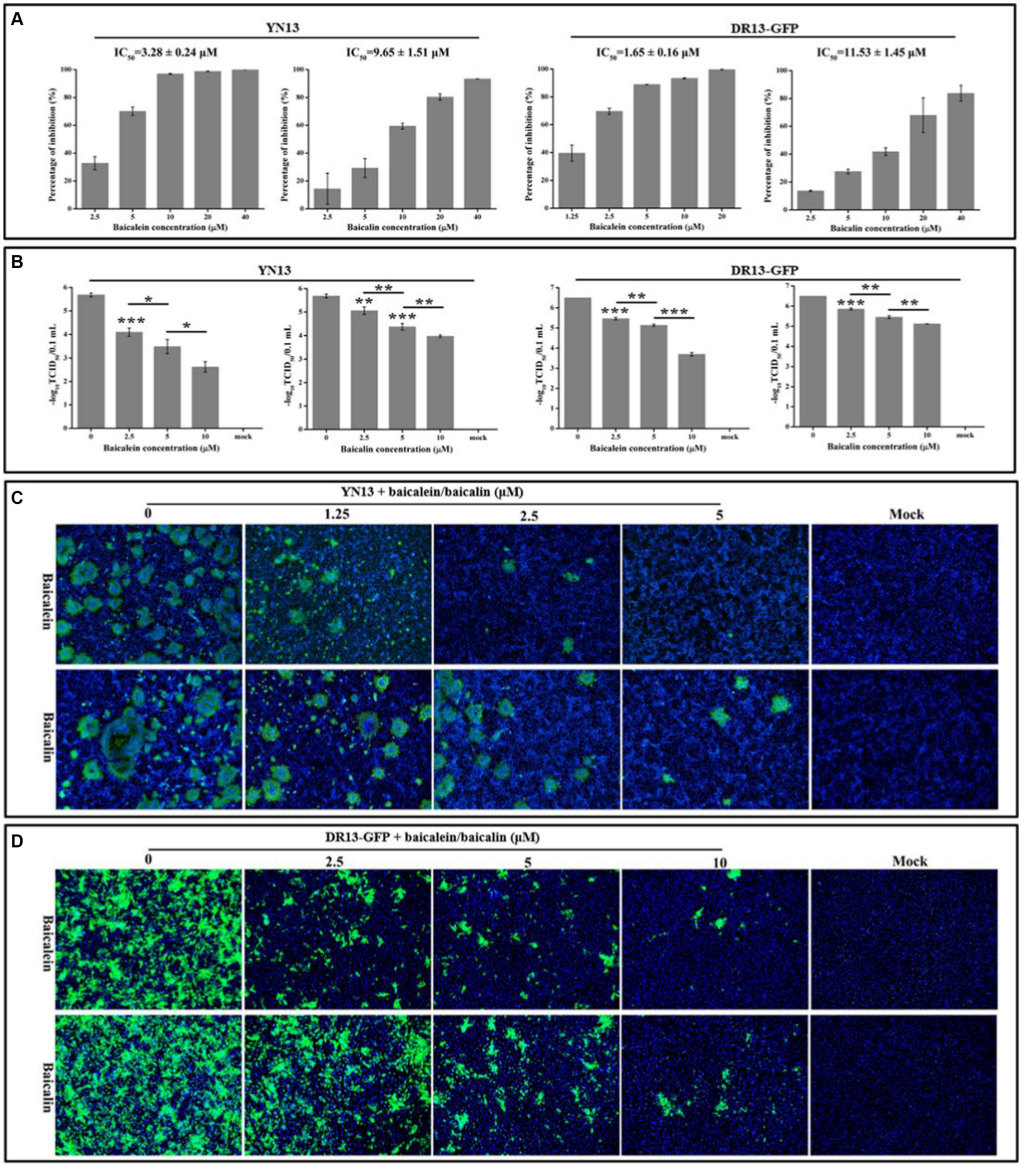
Antiviral activity of baicalein and baicalin against the PEDV YN13 strain and the DR13-GFP strain in Vero cells. **(A)** The inhibition ratio of baicalein and baicalin on the expression of PEDV M gene of PEDV YN13 strain and DR13-GFP strain. The IC_50_ was calculated by probit regression using the SPSS Statistics 17.0 software to assess the inhibition ratios at different inhibitor concentrations. **(B)** The inhibitory effects of baicalein and baicalin on the viral titer of PEDV YN13 strain and DR13-GFP strain. **(C)** The IFA analysis of the inhibitory effects of baicalein and baicalin on the expression of S protein in PEDV YN13 strain infected cells. **(D)** The fluorescence observation analysis of the inhibitory effects of baicalein and baicalin on the GFP expression in the DR13-GFP infected cells.

Real-time RT-PCR was employed to evaluate the antiviral properties of baicalein and baicalin on two PEDV strains. Baicalein displayed a dose-dependent inhibition of viral gene production in the YN13 and DR13-GFP strains of PEDV, with an IC_50_ of 3.28 ± 0.24 and 1.65 ± 0.16 μM, respectively (Figure 6A). Similarly, baicalin also demonstrated a dose-dependent inhibitory impact on viral gene production in the YN13 and DR13-GFP strains of PEDV, with an IC_50_ value of 9.65 ± 1.51 and 11.53 ± 1.45 μM, respectively (Figure 6A). Nonetheless, baicalein exhibited a stronger inhibitory effect than baicalin. The selectivity index (SI) of baicalein was found to be greater than 100 for both PEDV YN13 strain and DR13-GFP strain, while baicalin exhibited a selectivity index greater than 30 for both strains (Supplementary File S2). These findings suggest the potential of baicalein and baicalin as promising anti-PEDV agents.

Confirmatory evidence of the anti-PEDV effect of baicalein and baicalin in Vero cells was obtained using a TCID_50_ assay. Figure 6B depicts the significant reductions in titer of the YN13 and DR13-GFP strain that was achieved through the administration of baicalein or baicalin, in a dose-dependent manner. Moreover, the IFA was conducted to manifest the inhibitory effects of these two compounds on the quantities of YN13-infected cells. The IFA results demonstrated a dose-dependent reduction in the number of YN13-infected cells upon treatment with both baicalein and baicalin. Furthermore, the baicalein and baicalin-treated group exhibited a decrease in the size and number of syncytia, which is the characteristic CPE of YN13-infected cells (Figure 6C). This provided additional substantiation of their inhibitory effects on YN13 infection. DR13-GFP, a lab-engineered recombinant virus designed by substituting the ORF3 gene of DR13 strains with the GFP gene via the reverse genetic technique, allowed for the expression of GFP as an indicator of the inhibitory effects of anti-PEDV compounds on the quantities of viral-infected cells. Consistent with the IFA results, the quantities of the DR13-GFP strain infected cells decreased with increasing concentrations of baicalein and baicalin (Figure 6C).

### 3.7 The anti-PEDV effects of baicalein and baicalin in LLC-PK1 cells

**Figure 7:**
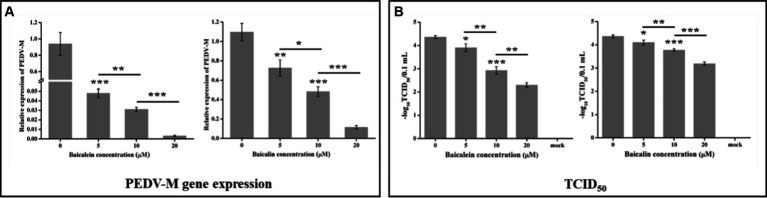
Antiviral activity of baicalein and baicalin against the PEDV YN13 strain in LLC-PK1 cells. LLC-PK1 cells grown on a 12-well plate were infected with the PEDV YN13 (MOI = 0.001) in the presence of baicalein and baicalin at different concentrations. Samples were collected at 24 hpi and subjected to quantify the viral gene by real-time RT-PCR **(A)** and measure the viral titer by TCID_50_ assay **(B)**.

To further confirm the anti-PEDV effects of baicalein and baicalin, another cell line, LLC-PK1, was selected. The results in the LLC-PK1 cells were similar to those in Vero cells. Both baicalein and baicalin were able to effectively suppress the replication of the YN13 gene and reduce the viral titers in a dose-dependent manner (Figure 7). Based on the data presented above, it can be concluded that baicalein and baicalin are effective inhibitors of PEDV infection *in vitro*.

### 3.8 The potential mechanisms for baicalein and baicalin suppressing PEDV infection

**Figure 8:**
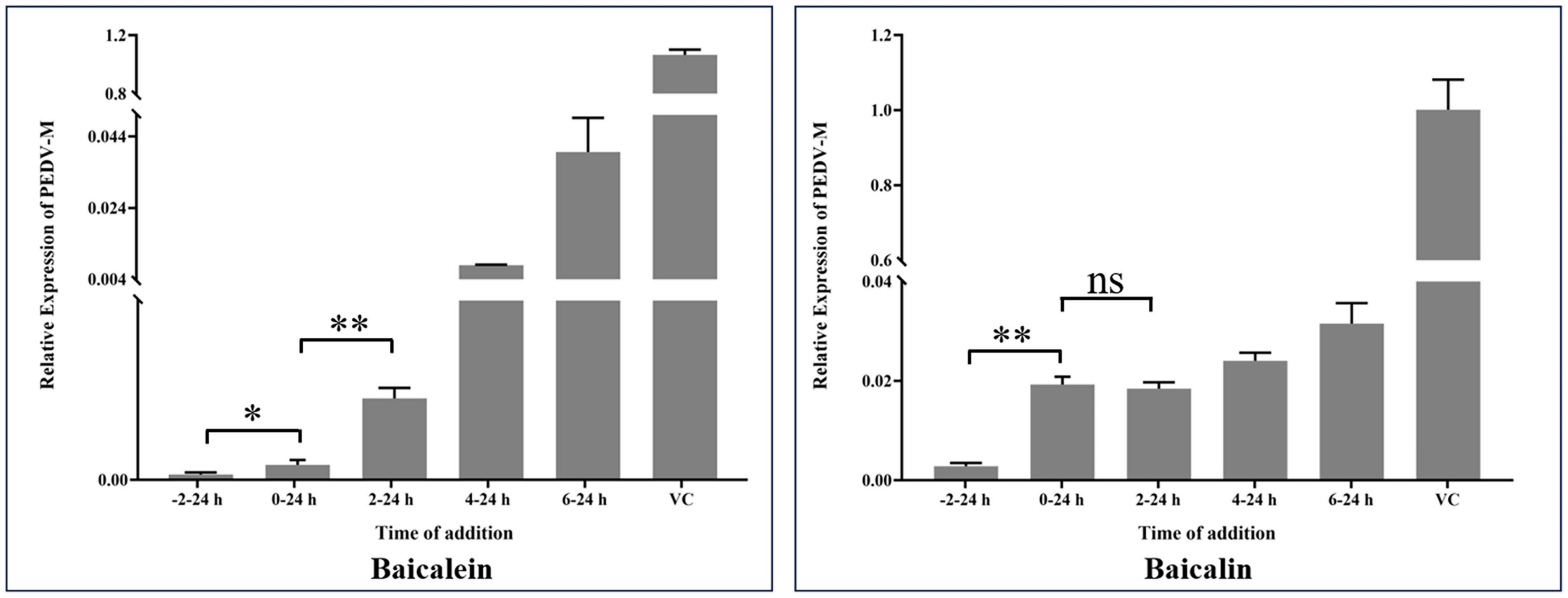
Time-of-addition effects of baicalein and baicalin on PEDV infection *in vitro*. Baicalein or baicalin (20 μM) was added to infected cultures either before (−2–24 h), during (0–24 h), or after (2–24 h, 4–24 h and 6–24 h) PEDV infection. At 24 hpi, samples were collected and subjected to calculate the expression of PEDV M gene by real-time RT-PCR. VC: virus control; Data are shown as means ± SD of three independent experiments, with the error bars representing the standard deviation. * represents a *p* value <0.05, ** represents a *p* value <0.01 and ns represents not significant.

Natural products seem to exhibit their antiviral effects through various mechanisms. To further explore the potential mechanisms of baicalein and baicalin in inhibiting PEDV infection *in vitro*, a time-of-addition experiment was conducted. Figure 8 demonstrates a significant difference between the −2-24 h group and the 0–24 h group, suggesting that pre-incubation with baicalein and baicalin prior to PEDV infection enhances their antiviral effects *in vitro*. It is widely recognized that viral attachment and invasion primarily occur within the first 2 h after PEDV infection. Notably, the baicalein treated group exhibited a significant difference between the 0–24 h group and the 2–24 h group, indicating that baicalein may influence the attachment or invasion step of PEDV. Conversely, baicalin treatment did not appear to affect PEDV attachment or invasion. Furthermore, the addition of baicalein and baicalin after PEDV infection (2–24 h, 4–24 h, and 6–24 h) also exhibited significant anti-PEDV effects, suggesting that these two molecules may exert their antiviral effects by interfering with viral replication within the host cells.

## 4 Discussion

PED is one of the major threats to the global swine industry and its primary harm is the high morbidity and mortality in piglets. While immunization with suitable vaccines can effectively prevent PED, the development of new vaccines has not kept up with the pace of PEDV mutation. Additionally, vaccine immunity does not protect piglets already infected with PEDV. Currently, there are no commercial drugs or therapeutic approaches available for treating PED. Therefore, there is an urgent need for novel anti-PEDV agents.

Target selection is one of the most important steps in the development of antiviral drugs. Viral proteases have been regarded as an important target for screening antiviral drugs. The conservation of viral proteases among different strains of a virus determines its suitability as an antiviral target. Although coronaviruses are RNA viruses with high variability, their 3CL^pro^ remains highly conserved. Furthermore, coronavirus 3CL^pro^ does not have similar homologous molecules in the host cells (Liu et al., 2020), making it an advantageous target for antiviral interventions. Therefore, the development of anti-PEDV drugs targeting 3CL^pro^ could become an appropriate strategy for preventing PED outbreaks.

Flavonoids are a significant class of natural products, as well as a type of polyphenolic plant secondary metabolites that are widely present in vegetables and fruits. Previous studies have demonstrated the ability of numerous flavonoid molecules to inhibit the activity of coronavirus 3CL^pro^. For instance, herbacetin, isobavachalcone, quercetin 3-β-d-glucoside, and helichrysetin were found to inhibit MERS-CoV 3CL^pro^ (Jo et al., 2019). Similarly, luteolin, apigenin, quercetin, amentoflavone, daidzein, puerarin, epigallocatechin, epigallocatechin gallate, gallocatechin gallate, and kaempferol were found to suppress SARS-CoV 3CL^pro^ activity (Ryu et al., 2010; Nguyen et al., 2012; Schwarz et al., 2014). Furthermore, myricetin, dihydromyricetin, and isodihydromyricetin were found to suppress SARS-CoV-2 3CL^pro^ activity (Su et al., 2021; Xiong et al., 2021). In our previous study, we also observed the effective inhibition of PEDV infection *in vitro* by quercetin, which targeted PEDV 3CL^pro^ (Li et al., 2020). Therefore, a flavonoid library was selected to screen for PEDV 3CL^pro^ inhibitors.

Although more than 30 flavonoids were screened from the library, only baicalein and baicalin were chosen for the subsequent anti-PEDV study. These two molecules were selected due to their low cost. In addition, baicalin is a glycoside compound composed of a molecule of baicalein and a molecule of glucuronic acid (Chen et al., 2014), making them as an ideal couple to study their inhibitory effects on PEDV 3CL^pro^ and viral infection. Previous studies have identified His41 and Cys144 as the active sites of PEDV 3CL^pro^. Furthermore, the S1 specificity pocket of PEDV 3CL^pro^, which consists of Phe139, Ile140, Asn141, Gly142, Ala143, Cys144, His162, Gln163, and Glu165, is crucial for substrate binding (Ye et al., 2016). To understand the binding mechanism between 3CL^pro^ and baicalein or baicalin, the active site residues and the S1 specificity pocket were selected as targets for molecule docking. Docking results (Figure 3) revealed that baicalein bound to His41 using its carbonyl group, as well as to Ile140, Gly142, Ala143, and His162 through its three phenolic hydroxyl groups. This suggests that the phenolic hydroxyl groups in baicalein play a significant role in the recognition and binding to PEDV 3CL^pro^. On the other hand, baicalin only bound to Asn141 and Gln163 using one hydroxyl group on the baicalein skeleton and the carboxy group in the glucuronic acid skeleton. Baicalin exhibited fewer interactions with amino acid residues on PEDV 3CL^pro^ compared to baicalein, possibly due to the masking effect of the glucuronic acid group on the hydroxyl groups of the baicalein skeleton and the resulting conformational change. The IC_50_ value of baicalein on PEDV 3CL^pro^ was significantly lower than that of baicalin, indicating that the presence of the glucuronic acid chemical group in baicalin reduces its binding affinity with the active sites of PEDV 3CL^pro^.

Two PEDV stains and two cell lines were selected to investigate the anti-PEDV effects of baicalein and baicalin. Our results demonstrated that baicalein and baicalin effectively inhibited PEDV replication at a micromolar range, and that their anti-PEDV effects were not influenced by the specific PEDV strain or cell line. The anti-PEDV effect of baicalein was superior to that of baicalin, possibly due to its stronger inhibitory effects on PEDV 3CL^pro^. As PEDV 3CL^pro^ is expressed once the viral genome enters the host cell, baicalein and baicalin are more likely to inhibit the early stage of PEDV replication. Figure 8 shows that adding baicalein and baicalin in the early stages of PEDV replication resulted in obvious antiviral effects, suggesting that their anti-PEDV effects may be related to their inhibitory effects on PEDV 3CL^pro^. Pre-treating cells with baicalein and baicalin enhanced their anti-PEDV effects, indicating the existence of other anti-PEDV mechanisms of these two molecules. Furthermore, baicalein was found to inhibit the attachment or invasion step of PEDV, although the detailed mechanisms still require further exploration.

The emerging highly virulent PEDV strains have been prevalent globally, and seriously affect the development of the pig farming industry. In our study, we identified baicalein and baicalin as two effective inhibitors of PEDV 3CL^pro^. Additionally, we discovered that baicalein and baicalin could serve as novel antiviral agents, potentially offering safe, effective, and affordable treatments for PEDV infection. Our findings will contribute to the development of new broad-spectrum antiviral drugs against PEDV.

## Data availability statement

The datasets presented in this study can be found in online repositories. The names of the repository/repositories and accession number(s) can be found in the article/Supplementary material.

## Author contributions

ZL: Conceptualization, Data curation, Funding acquisition, Methodology, Supervision, Writing – original draft, Writing – review & editing. LZ: Data curation, Investigation, Writing – original draft. LuW: Investigation, Writing – original draft. YiH: Investigation, Writing – review & editing. YZ: Investigation, Writing – review & editing. DZ: Data curation, Writing – review & editing. LeW: Methodology, Writing – review & editing. DY: Conceptualization, Funding acquisition, Writing – review & editing. YoH: Conceptualization, Methodology, Supervision, Writing – review & editing. TW: Conceptualization, Funding acquisition, Methodology, Supervision, Writing – review & editing.
