# Supplementary material for: Identification of two flavonoids antiviral inhibitors targeting 3C-like protease of porcine epidemic diarrhea virus
Source: Front Microbiol. 2024 Mar 20;15:1357470. doi: 10.3389/fmicb.2024.1357470 (PMC10987960; doi:10.3389/fmicb.2024.1357470)
Supplement: Supplementary file 1 [file Data_Sheet_1.PDF]

Compound Library Layout: Rack HYCPK26275

MCE® MedChemExpress

	1	2	3	4	5	6	7	8	9	10	11	12
A	Empty<br>A1	VialCode B2921069<br>Cat. No. HY-N6818<br>5, 7, 4'-Trimethoxyflavone<br>A2	VialCode B2921339<br>Cat. No. HY-N0460<br>1-Caffeoylquinic acid<br>A3	VialCode B2921155<br>Cat. No. HY-N6630<br>6-Methylflavone<br>A4	VialCode B2921043<br>Cat. No. HY-D0128<br>4-Methylherniarin<br>A5	VialCode B2921088<br>Cat. No. HY-N0533<br>4”-O-Glucosylvitexin<br>A6	VialCode B2921105<br>Cat. No. HY-N0625A<br>Alpinetin<br>A7	VialCode B2921134<br>Cat. No. HY-N1444<br>Complanatuside<br>A8	VialCode B2920992<br>Cat. No. HY-22024<br>5-Hydroxyflavone<br>A9	VialCode B2921002<br>Cat. No. HY-N4072<br>6''-O-Acetylglycitin<br>A10	VialCode B2921033<br>Cat. No. HY-N1910<br>4'-O-Methylbavachalcone<br>A11	Empty<br>A12
B	Empty<br>B1	VialCode B2921124<br>Cat. No. HY-N0578<br>Apigenin 7-glucoside<br>B2	VialCode B2921210<br>Cat. No. HY-N1201<br>Apigenin<br>B3	VialCode B2921304<br>Cat. No. HY-N0231<br>Bavachalcone<br>B4	VialCode B2921012<br>Cat. No. HY-N7058<br>cis- Jasmone<br>B5	VialCode B2921128<br>Cat. No. HY-N0509<br>Astilbin<br>B6	VialCode B2920995<br>Cat. No. HY-14595<br>Biochanin A<br>B7	VialCode B2921086<br>Cat. No. HY-N4167<br>3-O-Methylgalangin<br>B8	VialCode B2921380<br>Cat. No. HY-N0762<br>Isobavachin<br>B9	VialCode B2921118<br>Cat. No. HY-N0776<br>Isorhamnetin<br>B10	VialCode B2921145<br>Cat. No. HY-N0183<br>Formononetin<br>B11	Empty<br>B12
C	Empty<br>C1	VialCode B2921153<br>Cat. No. HY-107790<br>5-Methoxyflavone<br>C2	VialCode B2921039<br>Cat. No. HY-N0520<br>Calycosin-7-O-β-D-glucoside<br>C3	VialCode B2921106<br>Cat. No. HY-111928<br>5,7-Dimethoxyluteolin<br>C4	VialCode B2921102<br>Cat. No. HY-B1671<br>(+)-Kavain<br>C5	VialCode B2921078<br>Cat. No. HY-N1378<br>(E)-Cardamonin<br>C6	VialCode B2921312<br>Cat. No. HY-125130<br>Hesperetin 7-O-glucoside<br>C7	VialCode B2921212<br>Cat. No. HY-N0767<br>Isoorientin<br>C8	VialCode B2921341<br>Cat. No. HY-N4193<br>Glabrol<br>C9	VialCode B2921682<br>Cat. No. HY-N0731<br>Genkwanin<br>C10	VialCode B2921340<br>Cat. No. HY-N0374<br>Licochalcone C<br>C11	Empty<br>C12
D	Empty<br>D1	VialCode B2921451<br>Cat. No. HY-N4185<br>Licoflavone A<br>D2	VialCode B2921754<br>Cat. No. HY-N0768<br>Isoquercitrin<br>D3	VialCode B2921627<br>Cat. No. HY-15337<br>Hesperidin<br>D4	VialCode B2921333<br>Cat. No. HY-N0773<br>Isovitexin<br>D5	VialCode B2921170<br>Cat. No. HY-N0102<br>Isoliquiritigenin<br>D6	VialCode B2921215<br>Cat. No. HY-N0567<br>Hydroxysafflor yellow A<br>D7	VialCode B2921279<br>Cat. No. HY-N2131<br>Isosakuranetin<br>D8	VialCode B2921649<br>Cat. No. HY-N1458<br>Isoschaftoside<br>D9	VialCode B2921187<br>Cat. No. HY-N2600<br>Kuwanon H<br>D10	VialCode B2921386<br>Cat. No. HY-13425<br>Deguelin<br>D11	Empty<br>D12
E	Empty<br>E1	VialCode B2921545<br>Cat. No. HY-N0772<br>Isomangiferin<br>E2	VialCode B2921246<br>Cat. No. HY-N2117<br>Isoginkgetin<br>E3	VialCode B2921112<br>Cat. No. HY-N0393<br>Glabridin<br>E4	VialCode B2921642<br>Cat. No. HY-N0546<br>Ligustroflavone<br>E5	VialCode B2921317<br>Cat. No. HY-N4182<br>Licochalcone E<br>E6	VialCode B2921130<br>Cat. No. HY-N0125<br>Diosmetin<br>E7	VialCode B2921507<br>Cat. No. HY-N2445<br>Flavokawain C<br>E8	VialCode B2921126<br>Cat. No. HY-N6879<br>DiosMetin 7-O-β-D-Glucuronide<br>E9	VialCode B2921421<br>Cat. No. HY-N4296<br>Isosakuranin<br>E10	VialCode B2921229<br>Cat. No. HY-N0016<br>Glycitein<br>E11	Empty<br>E12
F	Empty<br>F1	VialCode B2921238<br>Cat. No. HY-N2068<br>Didymin<br>F2	VialCode B2921192<br>Cat. No. HY-N0257<br>Epimedin A<br>F3	VialCode B2921282<br>Cat. No. HY-N1510<br>Kaempferol 3-O-gentiobioside<br>F4	VialCode B2921236<br>Cat. No. HY-N2587<br>Irigenin<br>F5	VialCode B2921294<br>Cat. No. HY-N0258<br>Epimedin A1<br>F6	VialCode B2921241<br>Cat. No. HY-N7128<br>Flavanone<br>F7	VialCode B2921227<br>Cat. No. HY-N1939<br>Icariside I<br>F8	VialCode B2921176<br>Cat. No. HY-N0094<br>Ipriflavone<br>F9	VialCode B2921240<br>Cat. No. HY-N0636<br>Eriocitrin<br>F10	VialCode B2921571<br>Cat. No. HY-N6037<br>Gardenin B<br>F11	Empty<br>F12
G	Empty<br>G1	VialCode B2921161<br>Cat. No. HY-N4187<br>Licochalcone D<br>G2	VialCode B2921172<br>Cat. No. HY-N0452<br>Hyperoside<br>G3	VialCode B2921654<br>Cat. No. HY-N0260<br>Epmedin C<br>G4	VialCode B2921541<br>Cat. No. HY-N0713<br>Diosmetin-7-O-β-D-glucopyranoside<br>G5	VialCode B2921135<br>Cat. No. HY-N0268<br>Irisflorentin<br>G6	VialCode B2921479<br>Cat. No. HY-N1438<br>Hydroxygenkwanin<br>G7	VialCode B2921576<br>Cat. No. HY-N4247<br>Kuwanon G<br>G8	VialCode B2921539<br>Cat. No. HY-N2224<br>Guaijaverin<br>G9	VialCode B2921412<br>Cat. No. HY-N0765<br>Isoliquiritin<br>G10	VialCode B2921395<br>Cat. No. HY-N0628<br>Kaempferitrin<br>G11	Empty<br>G12
H	Empty<br>H1	VialCode B2921290<br>Cat. No. HY-N1950<br>Hispidulin<br>H2	VialCode B2921244<br>Cat. No. HY-N2286<br>Kushenol I<br>H3	VialCode B2921347<br>Cat. No. HY-15449<br>Kaempferide<br>H4	VialCode B2921206<br>Cat. No. HY-N4184<br>Licoflavone B<br>H5	VialCode B2921218<br>Cat. No. HY-N0778<br>Isorhamnetin-3-O-neohespeidoside<br>H6	VialCode B2921157<br>Cat. No. HY-N0259<br>Epimedin B<br>H7	VialCode B2921572<br>Cat. No. HY-N0436<br>Engeletin<br>H8	VialCode B2921680<br>Cat. No. HY-N0014<br>Icariin<br>H9	VialCode B2921583<br>Cat. No. HY-N0269<br>Echinatin<br>H10	VialCode B2921291<br>Cat. No. HY-N5024<br>Gambogenic acid<br>H11	Empty<br>H12

Compound Library Layout: Rack HYCPK26276

MCE® MedChemExpress

	1	2	3	4	5	6	7	8	9	10	11	12
A	Empty<br>A1	VialCode B0907248<br>Cat. No. HY-N2278<br>Kushenol A<br>A2	VialCode B3867681<br>Cat. No. HY-N6583<br>Licoflavonol<br>A3	VialCode B0907912<br>Cat. No. HY-N3389<br>Licoisoflavone A<br>A4	VialCode B1117823<br>Cat. No. HY-N2008<br>Luteolin 5-O-glucoside<br>A5	VialCode B0917851<br>Cat. No. HY-N2441<br>Methylophiopogonone A<br>A6	VialCode B2607503<br>Cat. No. HY-N7024<br>Quercetin 3-O-(6''-galloyl)-β-D-galactopyranoside<br>A7	VialCode B2921115<br>Cat. No. HY-N0577<br>Apiin<br>A8	VialCode B2921286<br>Cat. No. HY-110398<br>5,6,7-Trimethoxyflavone<br>A9	VialCode B2921213<br>Cat. No. HY-W006492<br>5a-Pregnane-3,20-dione<br>A10	VialCode B2921173<br>Cat. No. HY-N7108<br>7-Hydroxyflavone<br>A11	Empty<br>A12
B	Empty<br>B1	VialCode B2921287<br>Cat. No. HY-107198<br>(2S)-6-Prenylnaringenin<br>B2	VialCode B2921292<br>Cat. No. HY-Y0678<br>1,3,5-Trimethoxybenzene<br>B3	VialCode B2921308<br>Cat. No. HY-N2913<br>Ayanin<br>B4	VialCode B2921133<br>Cat. No. HY-N0222<br>Avicularin<br>B5	VialCode B2921297<br>Cat. No. HY-N2584<br>(2S)-Isoxanthohumol<br>B6	VialCode B2921163<br>Cat. No. HY-N0019<br>Daidzein<br>B7	VialCode B2921127<br>Cat. No. HY-N5106<br>(E)-Flavokawain A<br>B8	VialCode B2921799<br>Cat. No. HY-N4126<br>6-Demethoxytangeretin<br>B9	VialCode B2921108<br>Cat. No. HY-N7690<br>3,5,7,3′,4′-Pentamethoxyflavone<br>B10	VialCode B2921147<br>Cat. No. HY-N0898<br>Catechin<br>B11	Empty<br>B12
C	Empty<br>C1	VialCode B2921337<br>Cat. No. HY-N6629<br>3'-Methoxyflavonol<br>C2	VialCode B2921326<br>Cat. No. HY-N1457<br>Chrysosplenetin<br>C3	VialCode B2921214<br>Cat. No. HY-N0011<br>Baohuoside I<br>C4	VialCode B2921263<br>Cat. No. HY-N7632<br>5-Desmethylsinensetin<br>C5	VialCode B2921146<br>Cat. No. HY-N0519<br>Calycosin<br>C6	VialCode B2921185<br>Cat. No. HY-N0279<br>Cardamonin<br>C7	VialCode B2921139<br>Cat. No. HY-N2609<br>7,4'-Dihydroxyflavone<br>C8	VialCode B2921005<br>Cat. No. HY-N0233<br>Bavachin<br>C9	VialCode B2921300<br>Cat. No. HY-N1942<br>5-O-Demethylnobiletin<br>C10	VialCode B2921111<br>Cat. No. HY-N0015<br>Astragalin<br>C11	Empty<br>C12
D	Empty<br>D1	VialCode B2921372<br>Cat. No. HY-N2038<br>3, 5, 6, 7, 8, 3', 4'-Heptemthoxyflavone<br>D2	VialCode B2921365<br>Cat. No. HY-W011641<br>(±)-Naringenin<br>D3	VialCode B2921110<br>Cat. No. HY-N7030<br>5,7,3',4'-Tetramethoxyflavone<br>D4	VialCode B2921026<br>Cat. No. HY-N7199<br>(Rac)-Hydnocarpin<br>D5	VialCode B2921167<br>Cat. No. HY-N0234<br>Bavachinin<br>D6	VialCode B2921400<br>Cat. No. HY-128400<br>4'-Methoxychalcone<br>D7	VialCode B2921306<br>Cat. No. HY-121471<br>Chrysoeriol<br>D8	VialCode B2921201<br>Cat. No. HY-N0236<br>Corylin<br>D9	VialCode B2921180<br>Cat. No. HY-N0168A<br>(Rac)-Hesperetin<br>D10	VialCode B2921081<br>Cat. No. HY-Y1426<br>2'-Hydroxyacetophenone<br>D11	Empty<br>D12
E	Empty<br>E1	VialCode B2921230<br>Cat. No. HY-111802<br>3,4'-Dihydroxyflavone<br>E2	VialCode B2921008<br>Cat. No. HY-N0522<br>(-)-Gallocatechin gallate<br>E3	VialCode B2921316<br>Cat. No. HY-N0898A<br>(-)-Catechin<br>E4	VialCode B2921252<br>Cat. No. HY-N2376<br>Chrysin-7-O-glucuronide<br>E5	VialCode B2921220<br>Cat. No. HY-N6631<br>7-Methoxyisoflavone<br>E6	VialCode B2921003<br>Cat. No. HY-N0196<br>Baicalein<br>E7	VialCode B2921095<br>Cat. No. HY-N1881<br>4',5-Dihydroxyflavone<br>E8	VialCode B2921090<br>Cat. No. HY-N0540<br>Cynaroside<br>E9	VialCode B2921239<br>Cat. No. HY-N1454<br>Apigenin-7-glucuronide<br>E10	VialCode B2921047<br>Cat. No. HY-107818<br>4-Hydroxychalcone<br>E11	Empty<br>E12
F	Empty<br>F1	VialCode B2921057<br>Cat. No. HY-N2289<br>2''-O-Rhamnosylicariside II<br>F2	VialCode B2921113<br>Cat. No. HY-N0662<br>Amentoflavone<br>F3	VialCode B2921226<br>Cat. No. HY-N6963<br>Choerospondin<br>F4	VialCode B2921278<br>Cat. No. HY-N6020B<br>Butin<br>F5	VialCode B2921216<br>Cat. No. HY-N2208<br>4-Hydroxylonchocarpin<br>F6	VialCode B2921097<br>Cat. No. HY-N0516<br>Casticin<br>F7	VialCode B2921030<br>Cat. No. HY-N6628<br>6,2'-Dihydroxyflavone<br>F8	VialCode B2921779<br>Cat. No. HY-N1980<br>3'-Hydroxypuerarin<br>F9	VialCode B2921099<br>Cat. No. HY-14589<br>Chrysin<br>F10	VialCode B2921007<br>Cat. No. HY-N2145<br>4',7-Dimethoxyisoflavone<br>F11	Empty<br>F12
G	Empty<br>G1	VialCode B2921137<br>Cat. No. HY-111806<br>3,7,4'-Trihydroxyflavone<br>G2	VialCode B2921029<br>Cat. No. HY-N6596<br>7-Hydroxy-4H-chromen-4-one<br>G3	VialCode B2921107<br>Cat. No. HY-N0197<br>Baicalin<br>G4	VialCode B2921064<br>Cat. No. HY-136064<br>4,4'-Dimethoxychalcone<br>G5	VialCode B2921194<br>Cat. No. HY-N1978<br>3'-Methoxypuerarin<br>G6	VialCode B2921103<br>Cat. No. HY-N0526<br>2''-O-Galloylhyperin<br>G7	VialCode B2921109<br>Cat. No. HY-N0941<br>beta-Mangostin<br>G8	VialCode B2921041<br>Cat. No. HY-N0451<br>Acacetin<br>G9	VialCode B2921053<br>Cat. No. HY-N5011<br>5,7-Dimethoxyflavone<br>G10	VialCode B2921006<br>Cat. No. HY-N2144<br>7,4'-Di-O-methylapigenin<br>G11	Empty<br>G12
H	Empty<br>H1	VialCode B2921050<br>Cat. No. HY-N0897<br>Corylifol A<br>H2	VialCode B2921085<br>Cat. No. HY-N0001<br>(-)-Epicatechin<br>H3	VialCode B2921122<br>Cat. No. HY-N3017<br>Artemitin<br>H4	VialCode B2921100<br>Cat. No. HY-N2358<br>Blumeatin<br>H5	VialCode B2921276<br>Cat. No. HY-N4127<br>3'-Demethylnobiletin<br>H6	VialCode B2921189<br>Cat. No. HY-14615<br>[6]-Gingerol<br>H7	VialCode B2921119<br>Cat. No. HY-N1970<br>5,7-Dihydroxychromone<br>H8	VialCode B2921059<br>Cat. No. HY-N1993<br>5-Methyl-7-methoxyisoflavone<br>H9	VialCode B2921195<br>Cat. No. HY-N7110<br>6-Hydroxyflavone<br>H10	VialCode B2921293<br>Cat. No. HY-N7056<br>4'-Hydroxychalcone<br>H11	Empty<br>H12

Compound Library Layout: Rack HYCPK26277

MCE® MedChemExpress

	1	2	3	4	5	6	7	8	9	10	11	12
A	Empty<br>A1	VialCode B2921584<br>Cat. No. HY-N7368<br>Hibifolin<br>A2	VialCode B2921407<br>Cat. No. HY-N7176<br>Kaempferol 3-O-β-D-glucuronide<br>A3	VialCode B2921599<br>Cat. No. HY-N0382<br>Galangin<br>A4	VialCode B2921251<br>Cat. No. HY-13065<br>Isobavachalcone<br>A5	VialCode B2921104<br>Cat. No. HY-N6250<br>Isomucronulatol 7-O-glucoside<br>A6	VialCode B2921274<br>Cat. No. HY-N6896<br>Isoviolanthin<br>A7	VialCode B2921177<br>Cat. No. HY-N2497<br>Isoliquiritin apioside<br>A8	VialCode B2921144<br>Cat. No. HY-N0344<br>Farrerol<br>A9	VialCode B2921160<br>Cat. No. HY-N2279<br>Kurarinone<br>A10	VialCode B2921174<br>Cat. No. HY-N0528<br>Linarin<br>A11	Empty<br>A12
B	Empty<br>B1	VialCode B2921044<br>Cat. No. HY-N0112<br>Dihydromyricetin<br>B2	VialCode B2921681<br>Cat. No. HY-N2897<br>Dihydrokaempferol<br>B3	VialCode B2921771<br>Cat. No. HY-107207<br>Kaempferol 3-neohesperidoside<br>B4	VialCode B2921356<br>Cat. No. HY-107825<br>Flavonol<br>B5	VialCode B2921320<br>Cat. No. HY-126382<br>Hesperidin methylchalcone<br>B6	VialCode B2921409<br>Cat. No. HY-N0637<br>Eriodictyol<br>B7	VialCode B2921313<br>Cat. No. HY-N1949<br>Homoplantaginin<br>B8	VialCode B2921314<br>Cat. No. HY-N0678<br>Icaritin<br>B9	VialCode B2921136<br>Cat. No. HY-N0240<br>Herbacetin<br>B10	VialCode B2921324<br>Cat. No. HY-N0640<br>Kuromanin (chloride)<br>B11	Empty<br>B12
C	Empty<br>C1	VialCode B2921381<br>Cat. No. HY-N0018<br>Daidzin<br>C2	VialCode B2921548<br>Cat. No. HY-N0889<br>Ginkgetin<br>C3	VialCode B2921264<br>Cat. No. HY-14590<br>Kaempferol<br>C4	VialCode B2921733<br>Cat. No. HY-N0168<br>Hesperetin<br>C5	VialCode B2921049<br>Cat. No. HY-107569<br>Garcinol<br>C6	VialCode B2921198<br>Cat. No. HY-N0595<br>Genistin<br>C7	VialCode B2921585<br>Cat. No. HY-N0783<br>Eupatilin<br>C8	VialCode B2921804<br>Cat. No. HY-N0012<br>Glycitin<br>C9	VialCode B2921374<br>Cat. No. HY-N0530<br>Dryocrassin ABBA<br>C10	VialCode B2921338<br>Cat. No. HY-N2374<br>Eupatorin<br>C11	Empty<br>C12
D	Empty<br>D1	VialCode B2921168<br>Cat. No. HY-N0831<br>Jaceosidin<br>D2	VialCode B2921332<br>Cat. No. HY-N0875<br>IKarisoside A<br>D3	VialCode B2921458<br>Cat. No. HY-N0182<br>Fisetin<br>D4	VialCode B2921388<br>Cat. No. HY-N1941<br>Isosinensetin<br>D5	VialCode B2921456<br>Cat. No. HY-N3460<br>Isorhoifolin<br>D6	VialCode B2921018<br>Cat. No. HY-N2424<br>Flavone<br>D7	VialCode B2921401<br>Cat. No. HY-N0178<br>Diosmin<br>D8	VialCode B2921149<br>Cat. No. HY-14596<br>Genistein<br>D9	VialCode B3921715<br>Cat. No. HY-N0723<br>Neomangiferin<br>D10	VialCode B3921836<br>Cat. No. HY-129997<br>Luteolinidin (chloride)<br>D11	Empty<br>D12
E	Empty<br>E1	VialCode B3921419<br>Cat. No. HY-N2035<br>Moslosooflavone<br>E2	VialCode B3921783<br>Cat. No. HY-N0560<br>Oroxylin A<br>E3	VialCode B3920892<br>Cat. No. HY-N1504<br>Loureirin B<br>E4	VialCode B3921077<br>Cat. No. HY-N2119<br>Sciadopitysin<br>E5	VialCode B3920952<br>Cat. No. HY-N0779<br>Isosilybin<br>E6	VialCode B3921726<br>Cat. No. HY-13930<br>Miquelianin<br>E7	VialCode B3921622<br>Cat. No. HY-N2438<br>Methylophiopogonan one B<br>E8	VialCode B3921433<br>Cat. No. HY-N3006<br>Sakuranetin<br>E9	VialCode B3921213<br>Cat. No. HY-N0649<br>Narcissin<br>E10	VialCode B3921043<br>Cat. No. HY-N0752<br>Scutellarein<br>E11	Empty<br>E12
F	Empty<br>F1	VialCode B3921040<br>Cat. No. HY-N0703<br>Schaftoside<br>F2	VialCode B3920955<br>Cat. No. HY-N1968<br>Quercetin-3-O-β-D-glucose-7-O-β-D-gentiobiosiden<br>F3	VialCode B3921785<br>Cat. No. HY-N0119<br>Naringin Dihydrochalcone<br>F4	VialCode B3921822<br>Cat. No. HY-N1353<br>Rhamnocitrin<br>F5	VialCode B3921626<br>Cat. No. HY-N4136<br>Lonicerin<br>F6	VialCode B3921345<br>Cat. No. HY-N2127<br>Pinostrobin<br>F7	VialCode B3921424<br>Cat. No. HY-N0377<br>Liquiritigenin<br>F8	VialCode B3921595<br>Cat. No. HY-N2343<br>Procyanidin A2<br>F9	VialCode B3921412<br>Cat. No. HY-N0796<br>Procyanidin B2<br>F10	VialCode B3921035<br>Cat. No. HY-15097<br>Myricetin<br>F11	Empty<br>F12
G	Empty<br>G1	VialCode B3921150<br>Cat. No. HY-N2025<br>Oroxin A<br>G2	VialCode B3921191<br>Cat. No. HY-N1354<br>Reynoutrin<br>G3	VialCode B3921569<br>Cat. No. HY-N0873<br>Sagittatoside A<br>G4	VialCode B3920880<br>Cat. No. HY-N6673<br>Okanin<br>G5	VialCode B3921056<br>Cat. No. HY-N0148<br>Rutin<br>G6	VialCode B3921678<br>Cat. No. HY-N2572<br>Nepetin<br>G7	VialCode B3921788<br>Cat. No. HY-N3513<br>Mulberrin<br>G8	VialCode B3921273<br>Cat. No. HY-N0720<br>Neobavaisoflavone<br>G9	VialCode B3921616<br>Cat. No. HY-N3213<br>Naringenin triacetate<br>G10	VialCode B3921731<br>Cat. No. HY-N1377<br>Nevadensin<br>G11	Empty<br>G12
H	Empty<br>H1	VialCode B3921621<br>Cat. No. HY-N2123<br>Neoliquiritin<br>H2	VialCode B3921909<br>Cat. No. HY-N0155<br>Nobiletin<br>H3	VialCode B3921360<br>Cat. No. HY-N0100<br>Naringenin<br>H4	VialCode B3921057<br>Cat. No. HY-N0146<br>Quercetin (dihydrate)<br>H5	VialCode B3921654<br>Cat. No. HY-N0419<br>Quercimeritrin<br>H6	VialCode B3921480<br>Cat. No. HY-N0270<br>Ononin<br>H7	VialCode B3921676<br>Cat. No. HY-N4258<br>Panasenoside<br>H8	VialCode B3920895<br>Cat. No. HY-N0418<br>Quercitrin<br>H9	VialCode B3921804<br>Cat. No. HY-N4149<br>Quercetagetin<br>H10	VialCode B3921354<br>Cat. No. HY-N0162<br>Luteolin<br>H11	Empty<br>H12

Compound Library Layout: Rack HYCPK26278

	1	2	3	4	5	6	7	8	9	10	11	12
A	Empty<br>A1	VialCode B3921797<br>Cat. No. HY-N7036<br>Rhamnetin<br>A2	VialCode B3920941<br>Cat. No. HY-N0145<br>Puerarin<br>A3	VialCode B3921720<br>Cat. No. HY-N0804<br>Narirutin<br>A4	VialCode B3921796<br>Cat. No. HY-N0621<br>Morin<br>A5	VialCode B3921059<br>Cat. No. HY-N2562<br>Norwogonin<br>A6	VialCode B3921737<br>Cat. No. HY-N2344<br>Procyanidin A1<br>A7	VialCode B3921668<br>Cat. No. HY-N1318<br>Salvigenin<br>A8	VialCode B3921139<br>Cat. No. HY-N1505<br>Loureirin A<br>A9	VialCode B3921697<br>Cat. No. HY-N1435<br>Oroxin B<br>A10	VialCode B3921046<br>Cat. No. HY-N1413<br>Noricaritin<br>A11	Empty<br>A12
B	Empty<br>B1	VialCode B3921673<br>Cat. No. HY-N0376<br>Liquiritin<br>B2	VialCode B3921696<br>Cat. No. HY-N2437<br>Methylophiopogonanone A<br>B3	VialCode B3921558<br>Cat. No. HY-N0493<br>Pectolinarigenin<br>B4	VialCode B3921690<br>Cat. No. HY-N4314<br>Scutellarein tetramethyl ether<br>B5	VialCode B3921003<br>Cat. No. HY-N1549<br>Prunin<br>B6	VialCode B3921404<br>Cat. No. HY-N2481<br>Oroxylin A-7-O-glucuronide<br>B7	VialCode B3921604<br>Cat. No. HY-N2473<br>Methylnissolin-3-O-glucoside<br>B8	VialCode B3921255<br>Cat. No. HY-N0154<br>Neohesperidin dihydrochalcone<br>B9	VialCode B3921313<br>Cat. No. HY-N2342<br>Procyanidin C1<br>B10	VialCode B3921513<br>Cat. No. HY-N0428<br>Obacunone<br>B11	Empty<br>B12
C	Empty<br>C1	VialCode B3921322<br>Cat. No. HY-N1346<br>Robinin<br>C2	VialCode B3921007<br>Cat. No. HY-N0143<br>Phlorizin<br>C3	VialCode B3921006<br>Cat. No. HY-N4122<br>Neodiosmin<br>C4	VialCode B3921197<br>Cat. No. HY-N0575<br>Pinocembrin<br>C5	VialCode B3921025<br>Cat. No. HY-N0622<br>Morusin<br>C6	VialCode B3921803<br>Cat. No. HY-N4099<br>Luteolin-3-O-beta-D-glucuronide<br>C7	VialCode B3921051<br>Cat. No. HY-N1347<br>Robinetin<br>C8	VialCode B3921151<br>Cat. No. HY-N1463<br>Luteolin 7-O-glucuronide<br>C9	VialCode B3921305<br>Cat. No. HY-N1475<br>Nicotiflorin<br>C10	VialCode B3866864<br>Cat. No. HY-N0751<br>Scutellarin<br>C11	Empty<br>C12
D	Empty<br>D1	Empty<br>D2	Empty<br>D3	Empty<br>D4	Empty<br>D5	Empty<br>D6	Empty<br>D7	Empty<br>D8	Empty<br>D9	Empty<br>D10	Empty<br>D11	Empty<br>D12
E	Empty<br>E1	Empty<br>E2	Empty<br>E3	Empty<br>E4	Empty<br>E5	Empty<br>E6	Empty<br>E7	Empty<br>E8	Empty<br>E9	Empty<br>E10	Empty<br>E11	Empty<br>E12
F	Empty<br>F1	Empty<br>F2	Empty<br>F3	Empty<br>F4	Empty<br>F5	Empty<br>F6	Empty<br>F7	Empty<br>F8	Empty<br>F9	Empty<br>F10	Empty<br>F11	Empty<br>F12
G	Empty<br>G1	Empty<br>G2	Empty<br>G3	Empty<br>G4	Empty<br>G5	Empty<br>G6	Empty<br>G7	Empty<br>G8	Empty<br>G9	Empty<br>G10	Empty<br>G11	Empty<br>G12
H	Empty<br>H1	Empty<br>H2	Empty<br>H3	Empty<br>H4	Empty<br>H5	Empty<br>H6	Empty<br>H7	Empty<br>H8	Empty<br>H9	Empty<br>H10	Empty<br>H11	Empty<br>H12
