# Supplementary material for: Identification of two flavonoids antiviral inhibitors targeting 3C-like protease of porcine epidemic diarrhea virus
Source: Front Microbiol. 2024 Mar 20;15:1357470. doi: 10.3389/fmicb.2024.1357470 (PMC10987960; doi:10.3389/fmicb.2024.1357470)
Supplement: Supplementary file 2 [file Data_Sheet_2.PDF]

	CC_{50}[a] (μM)	IC_{50}[b] (μM)		SI[c]	
		YN13	DR13-GFP	YN13	DR13-GFP
Baicalein	> 400	3.28 ± 0.24	1.65 ± 0.16	> 113	> 220
Baicalin	> 400	9.65 ± 1.51	11.53 ± 1.45	> 35	> 30

Antiviral activity of baicalein and baicalin against PEDV in Vero cells.

a: Concentration required to reduce cell growth by 50%; b: Half maximal inhibitory concentration; c: Selectivity index = CC_{50}/IC_{50}.
